# Exploring the Challenges and Opportunities of ChatGPT in University Teaching for Health Sciences: A Narrative Review

**DOI:** 10.7759/cureus.94259

**Published:** 2025-10-10

**Authors:** Elena Arroyo-Bello, Beatriz González-Toledo, Jose Abad-Valle, Paloma Rodríguez-Gómez, Marta Garrigues-Ramón

**Affiliations:** 1 Nursing, Fundación Jiménez Díaz School of Nursing – Autonomous University of Madrid, Madrid, ESP; 2 Nursing, Health Research Institute – Fundación Jiménez Díaz University Hospital, Autonomous University of Madrid, Madrid, ESP

**Keywords:** artificial intelligence, chatgpt, higher education, impact, student

## Abstract

Among generative artificial intelligence (AI) tools, ChatGPT (OpenAI, San Francisco, CA, USA) has seen rapid adoption in the education sector due to its accessibility and versatility. While it supports a wide range of academic tasks, it also has significant limitations. Its difficulty in fully understanding the context of the conversations can lead to vague or inaccurate responses, raising questions about its reliability in educational and healthcare settings, where contextual accuracy is paramount. This narrative review aims to explore the challenges and prospects of using ChatGPT as a support tool in health science teaching and learning processes at the university level.

A review of the scientific literature on the use of generative artificial intelligence was conducted in the main databases between April and May of 2025. Studies published between January 2023 and March 27, 2025, were included to access the most up-to-date information available. The studies had to focus on the use of generative AI in higher education settings, specifically in the field of health sciences. A total of 18 documents met the inclusion criteria and were selected for analysis.

The results reveal that ChatGPT is widely used in undergraduate health education for tasks such as providing writing support, helping with content comprehension, generating quizzes, creating clinical simulations, and designing curricula. However, challenges such as misinformation, ethical concerns, and overreliance on AI were frequently noted. Additionally, disparities in access and lack of formal training for both students and educators were revealed to be significant barriers.

In conclusion, ChatGPT has significant potential to improve teaching and learning in health sciences education by providing personalized support, real-time feedback, and resource creation. However, effectively integrating ChatGPT into health sciences education requires paying special attention to ethical standards, equitable access, and developing digital literacy to ensure it complements, rather than replaces, fundamental human expertise.

## Introduction and background

Teaching is a profession that must always be up-to-date in terms of knowledge, methodology, and the technology used to improve the teaching and learning process for students. This need for updating arises due to the constant emergence of new knowledge, techniques, and methods of approaching learning in the classroom [1,2]. In this setting, emerging technologies have changed how knowledge is accessed, organized, and shared. The advent of interconnected resources, people, and content has engendered a paradigm shift in the realm of education, marked by enhanced efficiency and cost-effectiveness. Educational institutions have begun to adopt these tools as a complement to traditional methods, driven in part by the perception of usefulness, accessibility, and pedagogical value that their implementation promotes [3]. In this context of transformation, artificial intelligence (AI) is positioned as one of the most promising innovations, especially in the field of education, where it promises to reconfigure teaching and learning models through automated, personalized, and scalable solutions [4,5]. A significant proportion of educators recognize the necessity to modify their pedagogical and evaluative methodologies. The proposed changes are intended to promote critical thinking, ethical values, and the ability to adapt learning processes in environments that integrate AI [5].

Generative AI is a subdiscipline that uses pre-existing data to create new text, images, sounds, or video in response to user instructions, expanding the boundaries of creativity once considered exclusively human. Among these tools, ChatGPT, OpenAI's (San Francisco, CA, USA) generative pre-trained transformer (GPT)-based large language model (LLM), generates natural-language answers to complex prompts; its uptake in education has been rapid due to its accessibility and versatility. However, it has important limitations, including difficulty interpreting the full conversational context, which can lead to inaccurate or irrelevant outputs [6]. Beyond basic tasks (e.g., setting reminders), reported applications range from data analysis to decision support for clinical problems [2]. In the field of education, the potential of ChatGPT and related generative models lies in their ability to automatically generate customized learning materials and assessments, facilitating large-scale adaptive learning [5]. These models also offer immediate, continuous feedback, enabling instruction to be tailored to individual needs and overcoming time and space constraints [2,4]. Their thoughtful integration has encouraged innovative approaches such as the flipped classroom, promoting independent study in virtual environments, and the creation of dynamic, student-centered learning environments that support academic success [2].

In recent years, the field of AI has begun to transform various aspects of society, including higher education. Integrating it into teaching and learning has resulted in a significant transformation in the educational landscape. It enables the creation of customized virtual learning environments, driving teaching innovation and enhancing educational inclusivity and quality. However, its proper implementation requires careful planning and specialized training for teachers and students. In this regard, it has become a priority for universities to adapt their educational models to the social and industrial demands of the digital age. They are promoting digital skills among those involved in the educational system and using assessment tools that allow for the evaluation of the acceptance of these technologies [6]. Recent multinational evidence from Arab universities demonstrated that technology readiness and social influence positively predict educators’ perceptions of generative AI’s usefulness and effectiveness, whereas anxiety acts as a negative predictor [7].

Despite its advantages, the adoption of ChatGPT in education has raised concerns, particularly regarding its potential misuse for copying assignments and replacing human skills such as creativity and curiosity [2,8]. While AI can personalize learning and improve academic management, it also presents technical and ethical challenges. These include inaccuracies, cultural biases, and an inability to understand emotional context [9]. These issues underscore the importance of establishing ethical guidelines and ensuring equitable access to technology [8].

In the field of health sciences education, ChatGPT has demonstrated its capacity to produce accurate and contextualized responses to complex inquiries in a remarkably brief timeframe. This suggests its potential for transformative value, particularly in the interpretation of clinical data and the prediction of outcomes [10]. Disciplines such as medicine and nursing require specific training to equip future professionals with the skills to solve complex clinical problems. In this regard, leveraging AI-enhanced clinical simulation to enhance preparation for professional practice is a valuable strategy [2].

However, there are specific risks associated with this approach, including over-reliance on the tool, potential algorithmic bias that requires verification of results, and risks related to the privacy of clinical data due to the absence of clear regulatory frameworks [2,10]. To address these challenges, Ray, in 2024, has proposed a series of measures, including the promotion of collaboration between AI experts and healthcare professionals, the allocation of funding for specific research and pilot studies, the development of ethical guidelines, and the incorporation of content on AI in curricula [10]. A 2016-2022 systematic review by Crompton and Burke revealed that the use of AI in higher education experienced a substantial surge between 2021 and 2022, particularly within undergraduate programs [11]. Additionally, in 2024, Bond et al. underscored in their review the necessity for contemporary, pedagogically grounded evidence on conversational AI tools like ChatGPT within specific contexts [12]. This is particularly relevant in the context of university studies, especially for health science students, as they are laying the foundations of knowledge that will govern the future of their profession.

This review aims to explore the challenges and perspectives of using ChatGPT as a support tool in the teaching and learning processes of health sciences at the university level. Three questions are addressed. (1) How is ChatGPT currently being used in university health sciences classrooms, and where does it need improvement? (2) What challenges, risks, and ethical considerations accompany its use in this context? (3) What technical, pedagogical, and regulatory improvements are needed to ensure its responsible integration into educational environments?

## Review

Methods

Search Strategy

Between April and May 2025, we conducted a narrative review with a structured search on the use of generative artificial intelligence, specifically ChatGPT, using the Scopus, Web of Science, PubMed, Virtual Health Library, Dialnet, and Cochrane databases. The search strategy employed for this review involved the combination of key terms to identify relevant documents. The documents were filtered based on specific criteria, with the sections linked by the Boolean operator "AND." The first criterion focused on identifying documents related to AI, using terms such as "Artificial Intelligence," "Machine Learning," "ChatGPT," or "Generative AI" in the title. The second criterion targeted documents related to higher education, specifically those containing terms like "Higher Education," "University," "College," "Education," "Graduate Education," or "Student" in the title. Finally, the search was filtered based on the focus of the documents, whether it was the advantages, disadvantages, or benefits of using AI tools. To this end, terms such as "benefit," "advantage," "disadvantage," "challenge," "impact," or "perception" were used in the title, abstract, or keywords. Table 1 presents the different strategies used to search the select database.

**Table 1:** Search strategy applied to each of the databases

Database	Strategy
Web of Science (WoS)	TI=("Artificial Intelligence" OR "Machine Learning" OR "ChatGPT" OR "Generative AI") AND TI=("Higher Education" OR "University" OR "College" OR "Education" OR "Graduate Education" OR "Student") AND TS=("Benefit" OR "Advantage" OR "Disadvantage" OR "Challenge" OR "Impact" OR "Perception") and 2025 or 2024 or 2023 (Publication Years) and Article or Review Article (Document Types)
Scopus	TITLE ( ( "Artificial Intelligence" OR "Machine Learning" OR "ChatGPT" OR "Generative AI" ) AND ( "Higher Education" OR "University" OR "College" OR "Education" OR "Graduate Education" OR "Student" ) AND ( "Benefit" OR "Advantage" OR "Disadvantage" OR "Challenge" OR "Impact" OR "Perception" ) ) AND PUBYEAR > 2022 AND PUBYEAR < 2026 AND PUBYEAR > 2022 AND PUBYEAR < 2026 AND ( LIMIT-TO ( DOCTYPE , "ar" ) OR LIMIT-TO ( DOCTYPE , "re" ) ) AND ( LIMIT-TO ( SUBJAREA , "MEDI" ) OR LIMIT-TO ( SUBJAREA , "NURS" ) OR LIMIT-TO ( SUBJAREA , "DENT" ) OR LIMIT-TO ( SUBJAREA , "PHAR" ) )
Dialnet	Título: "ChatGPT" OR "Inteligencia artificial" AND (("educación superior" OR universidades OR universidad) AND (ventajas OR desventajas OR beneficios OR impacto OR percepción))- 2023-2025
PubMed	Search: ((("Artificial Intelligence"[Title] OR "Machine Learning"[Title] OR "ChatGPT"[Title] OR "Generative AI" [Title])) AND (("Higher Education"[Title] OR "University"[Title] OR "College"[Title] OR "Education"[Title] OR "Graduate Education"[Title] OR "Student" [Title]))) AND (("Benefit"[Title/Abstract] OR "Advantage"[Title/Abstract] OR "Disadvantage"[Title/Abstract] OR "Challenge"[Title/Abstract] OR "Impact"[Title/Abstract] OR "Perception" [Title/Abstract])) Filters: from 2023 - 2025 Sort by: Most Recent
Virtual Health Library	(ti:(( "Artificial Intelligence" OR "Machine Learning" OR "ChatGPT" OR "Generative AI" ) AND ( "Higher Education" OR "University" OR "College" OR "Education" OR "Graduate Education" OR "Student" ))) AND (( "Benefit" OR "Advantage" OR "Disadvantage" OR "Challenge" OR "Impact" OR "Perception" )) AND mj: ("Medical Education" OR "Medical Students" OR "Dental Education" OR "Nursing Education" OR "Medicine" OR "Doctors" OR "Nursing Students" OR "Dental Students") AND (year_cluster:[2023 TO 2025]) AND instance:"regional"
Cochrane	Record Title (( "Artificial Intelligence" OR "Machine Learning" OR "ChatGPT" OR "Generative AI" ) AND ( "Higher Education" OR "University" OR "College" OR "Education" OR "Graduate Education" OR "Student" )) AND Title Abstract Keyword ( "Benefit" OR "Advantage" OR "Disadvantage" OR "Challenge" OR "Impact" OR "Perception" )

Eligibility Criteria

Inclusion criteria: To ensure the most current and relevant information reflecting the present state of the topic, only studies published between January 2023 and March 27, 2025, were considered. The studies included in the review were required to focus specifically on the use of generative AI in higher education, particularly within health sciences. Additionally, the studies had to address the advantages, disadvantages, and limitations of generative AI tools, specifically ChatGPT, in university teaching and learning environments. The study exclusively encompasses research related to undergraduate education in health sciences.

Exclusion criteria: The exclusion of articles from consideration was determined by their relevance to health sciences education or to unrelated disciplines. Furthermore, opinion pieces, commentaries, and editorials lacking empirical evidence or methodological rigor were excluded. Studies that did not meet the minimum quality standards were excluded. These include studies lacking peer review or failing to meet the relevance criteria for the research focus.

Data Extraction and Selection

We used the software tool Rayyan (Rayyan Systems Inc., Cambridge, MA, USA) [13] to organize and filter records. Rayyan supports semi-automated title and abstract screening. The initial deduplication of articles and the facilitation of keyword-based document sorting were made possible by this platform [13]. Rayyan is a web-based application that helps researchers conduct systematic reviews more efficiently. It uses a predictive rating system that learns from reviewer decisions, thereby speeding up the selection process while maintaining quality [14].

The initial search identified a total of 649 articles in multiple databases: PubMed (n = 237), Virtual Health Library (n = 23), Web of Science (n = 285), Scopus (n = 51), Dialnet (n = 53), and Cochrane (no Cochrane review). After removing 73 duplicates in Rayyan, 576 articles remained for further selection. During this phase, another 303 articles were excluded through keyword-based filtering in Rayyan, as they did not meet the established inclusion criteria. Duplicates were removed using a combination of title matches, digital object identifier (DOI), and author-year. The documents were then reviewed, filtering them by keywords within Rayyan to facilitate decision-making. The keywords were aligned with the inclusion and exclusion criteria. Inclusion terms required explicit references to ChatGPT in higher education contexts relevant to undergraduate health sciences (medicine, nursing, dentistry, pharmacy, or related health fields and tasks such as teaching, learning, assessment, or simulation). Exclusion terms primarily targeted education at other higher or lower levels, other AI models, and disciplines unrelated to healthcare.

Following this initial selection, 273 documents were screened using ASReview (Utrecht University, NLD) [15], an open-source tool for AI-assisted systematic reviews. The ASReview uses active learning and machine learning models to prioritize relevant studies, reducing workload and improving decision-making speed [14]. The documents were reviewed by organizing them in descending order of priority while continuing to apply the same eligibility criteria, including or discarding documents manually, and following a non-automated review. At the end of this phase, 160 records were excluded, and 113 were promoted to full-text evaluation based on document type and relevance to undergraduate health sciences education.

To ensure a high-quality selection of peer-reviewed sources with transparent methods and analyzable data, the following documents were excluded: editorials, opinion pieces, letters, reflective essays, protocols, conference abstracts, and grey literature. This approach minimizes reliance on speculative assertions and ensures appraisal feasibility. In cases where the title or abstract was not conclusive, the full text was retrieved to determine eligibility.

**Figure 1:**
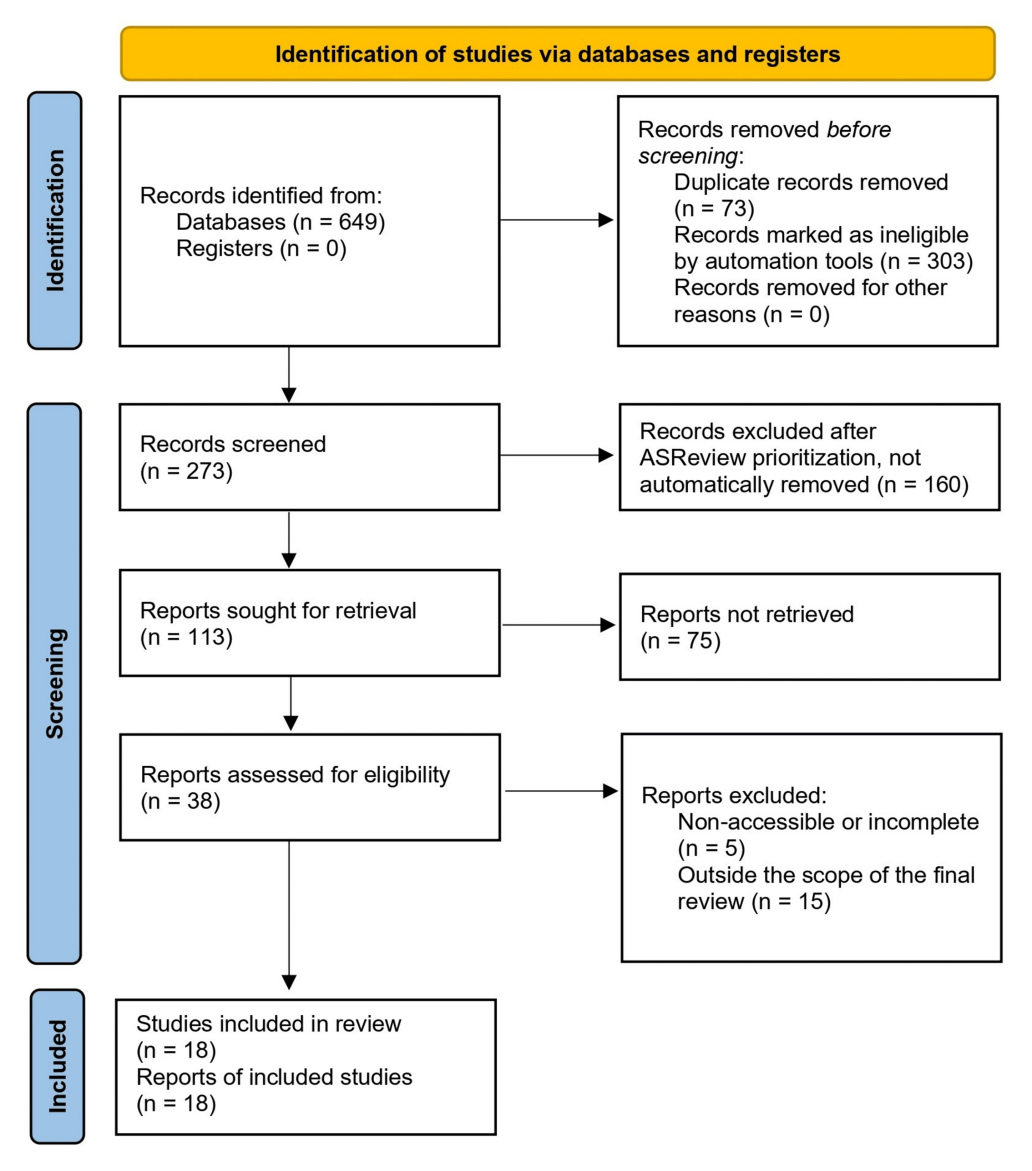
PRISMA flow diagram of the summarized search strategy n: Number of studies; PRISMA: Preferred Reporting Items for Systematic Reviews and Meta-Analyses

In the final screening, 38 documents were thoroughly reviewed, and 18 studies were selected for data analysis. It should be noted that no open access filter was applied. Full texts were included whenever they could be retrieved through institutional subscriptions; only five documents were excluded because the full text could not be obtained despite attempts to do so. Figure 1 outlines the search and selection strategy using the Preferred Reporting Items for Systematic Reviews and Meta-Analyses (PRISMA) 2020 flow diagram for new systematic reviews [16].

The methodological quality and risk of bias of the included studies were assessed by two independent reviewers using the Mixed Methods Appraisal Tool (MMAT). The MMAT is a validated instrument designed to assess a wide range of empirical study designs, including qualitative, quantitative (randomized, non-randomized, and descriptive), and mixed-methods studies. For each primary study, the five criteria corresponding to its specific design were applied. Studies were then scored based on the number of criteria met, providing a structured and comparable quality assessment [17]. Item-level judgments for primary studies are collated by design (qualitative, randomized controlled trial (RCT), quantitative descriptive, and mixed methods) in Appendix A.

However, the MMAT was not applicable to review articles. Instead, we used a review assessment framework based on established standards and best practices for narrative synthesis. This framework evaluated the clarity and justification of the review objective, the transparency and comprehensiveness of the search strategy, the definition and alignment of the inclusion and exclusion criteria, the document selection, extraction, and synthesis process, and the identification and management of methodological limitations and potential sources of bias. Ratings for the review articles are presented in Appendix B.

Results

After reviewing the documents, 18 studies related to the use of ChatGPT in university-level health sciences settings were included. All of these studies were published in high-impact English-language journals with different impact factors. Although no language restrictions were imposed during the review, the dominant language of the selected sources is English because most journals in the field of health sciences education are published primarily in English. Table 2 provides an overview of the sources, including the journal name, quartile (Q) classification (Q1, Q2, Q3, or Q4) based on the journal's impact factor per the SCImago Journal Rank (SJR) or Journal Citation Report (JCR), and the journal's corresponding 2024 impact factor, as well as the subject area of each journal.

**Table 2:** Overview of the included study sources Q: Quartile; JCR: Journal Citation Report; SJR: Scimago Journal Rank; JIF: Journal Impact Factor; ESCI: Emerging Sources Citation Index; SSCI: Social Sciences Citation Index; SCIE: Science Citation Index Expanded

Source name	Quartile	JIF or SJR	Edition (JCR)	Specific subject area
BMC Nursing	Q1 (JCR)	JIF 2024: 3.9	SSCI SCIE	Nursing
Nigerian Postgraduate Medical Journal	Q3 (JCR)	JIF 2024: 0.9	ESCI	Medicine: General and Internal
Al-Rafidain Journal of Medical Sciences	Q4 (SJR)	SJR 2024: 0.157	-	Medicine
Meta-Radiology	Q1 (SJR)	SJR 2024: 2.347	-	Computer-science health professions in medicine
Surgical and Radiologic Anatomy	Q3 (JCR)	JIF 2024: 1.2	SCIE	Radiology, nuclear medicine, medical imaging surgery, anatomy, and morphology
JMIR Medical Education	Q1 (JCR)	JIF 2023: 3.2	ESCI	Education, scientific disciplines
BMC Medical Education	Q1 (JCR)	JIF 2024: 3.2	SSCI SCIE	Education: Scientific disciplines and educational research
Digital Health	Q1-Q3 (JCR)	JIF 2024: 3.3	SSCI SCIE	Public health, environmental and occupational health policy, services medical informatics
European Journal of Dental Education	Q2 (JCR)	JIF 2024: 1.9	SCIE	Education, scientific disciplines: dentistry, oral surgery, and medicine
Postgraduate Medical Journal	Q1 (JCR)	JIF 2024: 2.7	SCIE	Medicine: General and internal
IEEE Access	Q2 (JCR)	JIF 2024: 3.6	SCIE	Telecommunications, computer science, information systems, and engineering
Journal of Medical Internet Research	Q1 (JCR)	JIF 2024: 6.0	SCIE	Health care sciences, services/medical informatics
Nurse Education Today	Q1 (JCR)	JIF 2024: 4.2	SSCI SCIE	Nursing, education, and scientific disciplines
Nurse Education in Practice	Q1 (JCR)	JIF 2024: 4.0	SSCI SCIE	Nursing
Teaching and Learning in Nursing	Q2 (JCR)	JIF 2024: 1.7	ESCI	Nursing, education, scientific disciplines

Of the 18 studies included in this review, seven were quantitative descriptive studies (38.88%), four were qualitative studies (22.22%), five were review articles (27.78%), one was a randomized controlled trial (5.56%), and one was a mixed-methods study (5.56%). Although no language restrictions were applied during the selection process, all included articles were published in English.

As part of the analysis, all references cited in the included reviews were screened to detect potential duplicates. Titles, authors, year of publication, journal, and DOI were compared to ensure accurate identification. Duplicated references between reviews were documented, and these were considered during synthesis to prevent counting them as independent sources and to avoid overestimating the available evidence.

The analysis revealed the presence of duplicated references among several of the included reviews (Table 3). The number of duplicates ranged from 9 to 28 references, representing between 15.38% and 31.37% of the total references within each review. No duplicated references were found between these reviews and the studies selected for the present work. Table 4 summarizes the main aspects collected from these studies, providing an overview of the findings and methodologies.

**Table 3:** Duplicate references identified among included reviews

Review	Duplicate references	Total references in review	Percentage of duplicates
Sahu et al. (2023) [18]	9	37	24.32%
Hamad et al. (2024) [19]	14	91	15.38%
Xu et al. (2024) [20]	16	51	31.37%
Shorey et al. (2024) [21]	28	132	21.21%
Gunawan et al. (2024) [22]	15	55	27.27%

**Table 4:** Overview of the included studies investigating the use of ChatGPT in university-level health sciences classrooms

Author and year	Title	Journal	Study design	Principal findings	Identified bias or limitations
Sahu et al. (2023) [18]	ChatGPT in research and health professions education: challenges, opportunities, and future directions	Postgraduate Medical Journal	Review	This review analyzed the educational and research applications of ChatGPT, emphasizing its potential to support personalized learning, clinical teaching, and academic writing. It identified key concerns, including ethical risks, academic dishonesty, and limitations in real-time accuracy. The authors advocate for institutional oversight and ethical guidelines. ChatGPT is seen as a supportive tool, not a replacement for educators.	Unclear search strategy; inclusion/ exclusion criteria not fully transparent.
Hamad et al. (2024) [19]	ChatGPT’s impact on education and healthcare: insights, challenges, and ethical consideration	IEEE Access	Review	This review analyzed 71 studies on ChatGPT's role in education and healthcare, identifying widespread applications in teaching, learning, clinical support, and medical research. It highlighted ChatGPT’s potential to enhance personalization, collaboration, and efficiency. The authors emphasized the importance of ethical, privacy, and accuracy considerations. They call for updated policies and ongoing evaluation to ensure responsible AI integration.	High transparency; no critical appraisal of included studies.
Xu, et al (2024) [20]	Current status of ChatGPT use in medical education: potentials, challenges, and strategies	Journal of Medical Internet Research	Review	This review outlines ChatGPT’s growing role in medical education, highlighting its benefits in personalized learning, curriculum design, and clinical scenario simulation. Key concerns include overdependence, academic integrity, and content accuracy. Ethical issues such as algorithmic bias and privacy risks are also emphasized. The authors propose strategies for responsible integration and model optimization to support medical training.	Reproducibility would be limited; insufficient detail in the selection methodology.
Shorey et al. (2024) [21]	A scoping review of ChatGPT's role in healthcare education and research	Nurse Education Today	Review	This scoping review synthesized literature on ChatGPT’s applications in healthcare education and research using a SWOT framework. The tool was praised for enhancing learning efficiency, accessibility, and engagement. Concerns included reliability, ethical risks, and the potential erosion of critical thinking. The authors recommend regulated, pedagogically sound implementation strategies.	No critical appraisal of included studies; exclusion of grey literature; potential subjectivity in SWOT-based synthesis.
Gunawan et al. (2024) [22]	ChatGPT integration within nursing education and its implications for nursing students: a systematic review and text network analysis	Nurse Education Today	Review	This systematic review and text network analysis identified four key themes in the literature: academic writing, healthcare simulation, data modeling, and personal development. Sentiment analysis revealed a mostly positive view of ChatGPT's role in nursing education. The study emphasized ChatGPT's transformative potential for skill-building and simulation-based learning. The authors advocate for responsible integration, supported by ethical data practices and cross-sector collaboration.	No quality assessment of included studies; diverse article types.
Salama et al. (2025) [23]	Knowledge, attitudes, and practices toward AI technology (ChatGPT) among nursing students at Palestinian universities	BMC Nursing	Quantitative descriptive	The study revealed that while nursing students in Palestine hold favorable views toward AI and support its integration into education, most lack formal training and practical experience with tools like ChatGPT. While its academic usefulness is widely acknowledged, concerns persist regarding its accuracy and the potential for replacing healthcare professionals. Key barriers include limited curricula, training, and infrastructure. The authors advocate for structured AI education to equip students with the necessary skills for careers in tech-integrated healthcare.	Convenience sampling; no control for nonresponse bias.
Oluwadiya, et al (2023) [24]	Exploring artificial intelligence in the Nigerian medical educational space: an online cross-sectional study of perceptions, risks, and benefits among students and lecturers from ten universities	Nigerian Postgraduate Medical Journal	Quantitative descriptive	This study found moderate AI knowledge among Nigerian medical students and lecturers, with students slightly ahead. While attitudes were positive, formal training and the use of tools like ChatGPT were limited. Students expressed more concern over AI risks. The authors call for structured AI education and ethical guidelines.	Voluntary online participation; absence of randomization introduces selection bias.
Mohammed et al. (2024) [25]	Perceptions of senior pharmacy students towards the impact of artificial intelligence on university education and scientific writing: a qualitative study	Al-Rafidain Journal of Medical Sciences	Qualitative	Senior Iraqi pharmacy students frequently used ChatGPT for assignments and understanding complex topics. While they valued its speed and efficiency, many expressed concerns about declining writing skills and reduced motivation to search for information. Some worried about misinformation and overdependence. The authors urge educators to adapt teaching strategies to preserve students' critical thinking and originality.	Potential recall and interviewer bias; culturally situated interpretations.
Song et al. (2024) [26]	Integrating AI in college education: positive yet mixed experiences with ChatGPT	Meta-Radiology	Quantitative descriptive	This study assessed ChatGPT integration into a medical imaging course, revealing high student engagement and preference for customized versions like ChatGe-V2. While most valued its interactivity and speed, concerns remained over accuracy and trust in responses. Use varied significantly depending on the instructor's encouragement. The authors suggest personalized AI tools can support learning but require ongoing evaluation.	Small sample from two course sections; limited external validity.
Totlis et al. (2023) [27]	The potential role of ChatGPT and artificial intelligence in anatomy education: a conversation with ChatGPT	Surgical and Radiologic Anatomy	Quantitative descriptive	This study evaluated ChatGPT-4’s potential as a supplementary tool in anatomy education using an interview-based assessment. The model demonstrated the ability to generate structured content, clinically relevant explanations, and formative assessments, though minor factual inaccuracies were noted. The authors conclude that while ChatGPT shows promise, it should serve as an adjunct to, not a substitute for, formal instruction.	No human participants; risk of evaluator subjectivity.
Cherrez-Ojeda, et al (2024) [28]	Understanding health care students’ perceptions, beliefs, and attitudes toward AI-powered language models: cross-sectional study	JMIR Medical Education	Quantitative descriptive	This survey of 2,661 health-profession students examined ChatGPT’s use, perceived knowledge, risks, ethics, and attitudes, finding 42.99% unaware and a median knowledge of 2.00, with neutral ethical views and generally positive attitudes. Among users, common uses were homework (70.97%), research papers (41.94%), and health education (22.98%); 81.17% wanted further training, but lack of time (46.37%) was the main barrier.	Convenience sampling; lack of nonresponse tracking.
Orok et al. (2024) [29]	Pharmacy students' perception and knowledge of chat-based artificial intelligence tools at a Nigerian University	BMC Medical Education	Quantitative descriptive	This study found that Nigerian pharmacy students exhibited high familiarity and generally positive perceptions toward chat-based AI tools, particularly ChatGPT. Use was highest for assignments and study support, especially among students with prior AI training. Misconceptions about AI's capabilities and accuracy were common. The authors recommend integrating structured AI education into the pharmacy curriculum to enhance literacy and ethical use.	No major limitations identified; random sampling and instrument validation confirmed.
Veras et al. (2024) [30]	A mixed methods crossover randomized controlled trial exploring the experiences, perceptions, and usability of artificial intelligence (ChatGPT) in health sciences education	Digital Health	Randomized controlled trial (RCT)	This study assessed ChatGPT-3.5’s usability and perceived effectiveness among health sciences students through a mixed-methods crossover RCT. Findings indicate that students found ChatGPT more intuitive and useful than traditional tools, particularly for brainstorming and productivity. However, concerns about academic integrity, misinformation, and ethical usage were prominent. The authors emphasize the need for institutional policies and clearer guidelines to responsibly integrate AI into educational contexts.	Lack of allocation concealment; no mention of intention-to-treat analysis.
Uribe et al. (2024) [31]	Artificial intelligence chatbots and large language models in dental education: worldwide survey of educators	European Journal of Dental Education	Quantitative descriptive	A global survey of 428 dental educators revealed growing interest in AI chatbots like ChatGPT, particularly for knowledge acquisition, research, and clinical decision-making. Perceptions varied regionally, with higher enthusiasm in Africa, Asia, and the Americas. Educators emphasized the need for training and clear implementation guidelines. The study concludes that responsible AI integration can enrich dental education if pedagogical and ethical challenges are addressed.	Low response rate (28%), possible selection bias.
Moskovich et al. (2025) [32]	Health profession students' perceptions of ChatGPT in healthcare and education: insights from a mixed-methods study	BMC Medical Education	Mixed methods	This mixed-methods study found that health profession students in Israel generally viewed ChatGPT positively, especially for information retrieval and academic support. Usage was moderate, with students appreciating its efficiency but expressing concerns over reliability and ethical implications. Across disciplines, perceptions were consistent. The authors recommend structured training and clear guidelines to support the responsible integration of ChatGPT in health education.	No significant bias noted; integrated methods and design robustness reported.
Sarikahya et al. (2025) [33]	The impact of ChatGPT on nursing education: a qualitative study based on the experiences of faculty members	Nurse Education Today	Qualitative	This qualitative study explored nursing faculty members’ experiences with ChatGPT, identifying its benefits for teaching efficiency, content generation, and information access. Faculty emphasized its support for theoretical learning and professional development. Concerns included academic integrity, content verification, and effects on students’ critical thinking. The authors recommend structured AI literacy and integration strategies in nursing education.	No major bias; triangulation and methodological rigor applied.
Summers et al. (2024) [34]	Navigating challenges and opportunities: nursing students' views on generative AI in higher education	Nurse Education in Practice	Qualitative	This qualitative study explored nursing students’ perceptions of generative AI, revealing mixed views shaped by ethical concerns, equity, and practical use. Students valued AI’s potential for enhancing productivity and learning, but feared its impact on critical thinking and academic integrity. Generational differences influenced acceptance and usage patterns. The authors recommend structured, ethical integration of AI into nursing education.	Social desirability bias; students may have responded positively due to expectations.
Ahmed et al. (2024) [35]	AI in higher education: unveiling nursing students' perspectives on ChatGPT's challenges and opportunities	Teaching and learning in nursing	Qualitative	This qualitative study explored Emirati nursing students’ experiences with ChatGPT, revealing both enthusiasm for its flexibility and support in learning and concerns about academic integrity and overreliance. Students viewed ChatGPT as a helpful tutor and information tool, but questioned its trustworthiness and ethical use. Their insights emphasized the need for institutional guidelines and digital literacy training. The authors recommend structured integration to promote responsible use of AI in nursing education.	Risk of participant reactivity and interviewer influence.

After a thorough review of the documents, the relevant results are presented, answering the three key questions outlined at the beginning of this review.

How Is ChatGPT Currently Being Used in University Health Sciences Classrooms, and Where Does It Need Improvement?

In university-level health sciences classrooms, ChatGPT is one of the most popular generative AI programs among students [25,31]. It is mainly used for academic writing tasks, such as summarizing content, perfecting grammar, and developing arguments for papers and research projects [29], as well as for studying and completing assignments, reflecting its integration into daily academic routines [29,32]. Pharmacy and medical students, for example, frequently use it to understand complex topics in subjects related to therapeutics, biotechnology, and chemistry [25]. Students particularly value its instant and interactive explanations for unraveling complex content. When customized versions trained with materials from a specific course were used, student ratings proved to be better than generic AI and, in certain items, even better than responses from teaching assistants, according to peer comparisons [26].

It is also used for self-directed learning and exam preparation, generating interactive quizzes, providing real-time explanations of anatomical structures, and simulating patient cases [27,31]. This is particularly evident in more intricate subjects such as the vascular system and neural pathways. In 2023, Totlis et al. described the use of ChatGPT in anatomy courses, simulating clinical cases in which students trace nerve pathways and explain musculoskeletal functions step by step [27].

In the educational sector, ChatGPT finds primary application in personalized learning, teacher support, clinical simulation design, and assistance with academic writing and data analysis [21]. In the field of curriculum and instructional design, ChatGPT has the potential to streamline lesson development, assessment design (including rubrics), and feedback generation [19,20,33]. This support is a significant asset for novice teachers, as it facilitates planning and content creation [19,33].

ChatGPT has also been integrated into simulation-based learning environments, allowing students to practice clinical reasoning through AI-generated patient scenarios involving medication management and emergency care [22]. Additionally, ChatGPT is incorporated into clinical training through role-playing and virtual simulations, assisting students in honing their reasoning abilities and engaging with patient care scenarios in controlled environments [18,20].

It has been found to be an intuitive and easy-to-use program by educators and students alike, with positive impacts on productivity and classroom learning [30,34,35]. The accessibility of the platform, which is available 24/7, and its real-time responsiveness are particularly valuable for self-paced learning outside of traditional class hours. This feature saves time and promotes student autonomy, which reduces stress, especially for anxious or introverted students [32,35]. It also facilitates language translation and personalized tutoring for non-native English speakers, expanding its reach as a teaching aid in multilingual classrooms [31,33]. This supports the idea that ChatGPT is a valuable resource for disadvantaged and multilingual student populations, as it improves access to educational materials and reduces barriers to comprehension [18,19,29,31]. Furthermore, it supports both individual and collaborative learning, making it a versatile resource in various academic contexts [29].

Mohammed et al. discussed the use of ChatGPT by pharmacy seniors to help them complete their final projects and facilitate the translation of documents during the research and writing processes [25]. Their applications include assisting with manuscript writing, summarizing bibliographies, and creating structured outlines. They help students build logical academic arguments, refine their language, and navigate the publication process [22].

Despite its advantages, there are areas that need improvement. One of these is increasing the rigor and accuracy of the system in clinical and basic domains. Evidence in anatomy shows that, although ChatGPT offers clear and useful descriptions and generates study materials, it can fail with anatomical variants or fine details, so it should not be considered the sole source or a substitute for the teacher. A notable limitation of ChatGPT is the absence of visual aids such as diagrams, a crucial element for visual disciplines such as this one [27]. Students and teachers have identified inconsistencies, hallucinations, and a lack of precision in specialized topics [21,26], as well as a lack of ability to discriminate information [19,20,25-27,34,35].

However, various studies with university students and teachers, respectively, have found that the tool is beneficial but does not inherently improve critical thinking or writing skills. These studies have recommended a re-evaluation of teaching and assessment strategies [25,30]. This aspect suggests that excessive use may potentially result in a decline in critical thinking, creativity, and independent learning [18,25,29,32,34,35]. Students have expressed concerns that the frequent use of ChatGPT for academic assignments and study may potentially lead to a decline in their writing skills and the ability to locate information from primary scientific sources [25]. This could potentially compromise critical academic abilities such as analytical reasoning and research literacy [18,32]. Orok et al. reported that many students mistakenly believed that AI-generated data is always accurate and that AI is designed to replace traditional teaching methods. These misconceptions may lead to a reliance on AI, which could potentially disrupt the core principles of health education [29].

Additionally, the absence of emotional intelligence and human interaction is identified as a limitation. ChatGPT is a powerful tool for data processing and knowledge transfer, but it has its limitations when it comes to empathy, nuance, and therapeutic communication skills, which are vital in fields such as education and healthcare [18-20,22,25,32-34].

What Challenges, Risks, and Ethical Considerations Accompany Its Use in This Context?

The available evidence indicates that integrating these tools carries significant risks and ethical considerations, including insufficient training, which establishes the starting point: Oluwadiya et al. reported that only a small proportion of students (4.0%) and teachers (1.4%) had received formal training in AI, and all had done so independently, without institutional support. Therefore, the integration of ChatGPT in classrooms is more incidental than strategic. This indicates an urgent need for structured, AI-based pedagogy [24]. In a similar vein, Salama et al. (2025) noted that 69.9% of nursing students lacked specific training in ChatGPT, despite their favorable attitudes toward its incorporation into the curriculum [23].

The absence of training has led to concerns regarding the lack of clear guidelines for implementing its use. Uribe et al. highlighted that 59.3% of educators cited inadequate support or training as an obstacle [31]. Oluwadiya et al. reported that 70.6% of students and 60.8% of teachers expressed concern that AI would dehumanize healthcare. Most believed that AI could diminish the clinical skills of physicians (79.3% of students; 71.3% of teachers) and, to a lesser extent, that physicians could become obsolete (57.6% of students; 34.7% of teachers) [24]. However, merely utilizing ChatGPT does not ensure AI literacy; it demands a curriculum that incorporates explicit instruction on response evaluation, source traceability, biases, privacy, and security, thereby transforming it into an interdisciplinary learning experience [20].

Another important aspect to consider is that not all students use it, or have never used it, in high percentages [23,24]. This underscores the importance of ensuring equity and access to new tools. If some students have access to AI while others do not, it creates an unfair advantage and raises concerns about the fairness of training and assessment processes [20,21,30]. Additionally, generational and socioeconomic differences in AI knowledge further exacerbate disparities, as younger, more technologically proficient students tend to perform better [20,23,34], and males tend to outperform females [23].

Cultural biases and limitations are another serious concern. ChatGPT's outputs are largely determined by Western-centric training data, which can perpetuate biased perspectives and marginalize diverse worldviews [21,22,28]. Bias and inclusivity pose additional challenges. ChatGPT has been shown to reflect Western-centric biases and lack cultural sensitivity, which may limit its usefulness in diverse or global healthcare education contexts [20,22,28,31]. To address this issue, models must be adjusted using culturally relevant and domain-specific datasets to improve equity, safety, and contextual appropriateness [20,21].

While ChatGPT offers numerous educational benefits, some students have reported that it disrupts their focus. Specifically, 65.7% of students indicated that using AI tools such as ChatGPT during class made it difficult for them to stay focused on the lesson, and 48.2% believed that using ChatGPT during online classes increased the likelihood of being distracted by their home environment. These findings suggest that while ChatGPT can be a valuable learning aid, its use during live or remote sessions may unintentionally reduce student attention and concentration, which could affect the quality of the learning experience [29].

When considering ethical concerns, the primary challenge is academic dishonesty and plagiarism. The capacity of ChatGPT to generate polished and coherent responses prompts inquiries into authorship, originality, and fair assessment [18-20,25,29]. Some institutions have reported an increase in students submitting AI-generated work without disclosing it. Educators emphasize that current plagiarism detection tools are inadequate for identifying AI-produced content [18]. Additionally, students have voiced concerns regarding potential sanctions for the use of AI tools without explicit guidelines. They have underscored the significance of inclusive and transparent institutional strategies to address these concerns [32]. 

Another ethical preoccupation frequently reiterated in the literature on the use of ChatGPT in education and health is data privacy [18,19,22,35]. It has been noted in several studies that the current models are not adequately equipped to handle sensitive information. This may result in a compromise of the confidentiality of patients and students, exposing them to risks of misuse, fraud, or leaks [18,22]. The necessity for clear regulatory frameworks and robust security measures is emphasized as crucial to prevent unauthorized sharing of information. It is imperative to emphasize that any integration of ChatGPT must include mechanisms for informed consent and institutional accountability. A breach of privacy not only carries legal consequences but also erodes trust in AI-based educational and healthcare solutions [19].

What Technical, Pedagogical, and Regulatory Improvements Are Needed to Ensure Its Responsible Integration Into Educational Environments?

In terms of technical skills, models need to be adjusted with culturally relevant and field-specific datasets to improve equity, safety, and disciplinary appropriateness [20-22,28,31]. Concurrently, data protection and traceability safeguards must be implemented in simulated teaching and clinical environments: rules of use, clear consents and technical limits, recording the use of AI, and ensuring proper ethical oversight and responsible reporting [19,30,35]. These technical measures are complemented by periodic usability assessment and review cycles [30].

Given that the use of ChatGPT does not inherently promote higher-order skills (e.g., critical analysis, ethical reasoning, creativity) and may encourage surface-level learning habits, it is recommended that pedagogical reforms and evaluative redesigns focus on critical thinking and AI-aware pedagogy [18,19,25,31,32]. This change requires institutional support [19,24,29-31,33,35]. However, when it is integrated purposefully, i.e., with a focus on case design, evidence-based argumentation, and guided feedback, improvements in cross-cutting skills relevant to professional practice are observed. To avoid replicating biases, such as cultural ones, it should be noted that any integration in the use of this tool must be accompanied by data curation of sources, stratified evaluation by linguistic and cultural groups, and transparent governance regarding the limits and origin of the information [19].

At the pedagogical level, one of the most important areas for improvement is equity in the use of AI tools, which depends on universal access. The proposed solutions are to guarantee equal access and regulate the use of AI tools to prevent plagiarism and dependency [34]. For this purpose, it is essential to promote digital literacy among students and teachers [24]. This would enable them to utilize ChatGPT in a deliberate and mindful manner. It is important to recognize the misconceptions surrounding the role of AI, as confusion persists in identifying applications that are not AI. This reflects practical familiarity but a lack of conceptual clarity. The integration of AI literacy into educational curricula should encompass a comprehensive understanding of the mechanisms, limitations, and ethical dimensions of these tools [18,24,29].

Ensuring equity in access is paramount for maintaining fairness in training and assessment. Solutions must include provisions for equal access and effective regulation to prevent plagiarism and dependency [34]. These considerations extend to infrastructure, training, and pedagogical support, with regional variations in the perception of the potential of chatbots and concerns about reduced human interaction, which demands equitable resources [23,31].

Data protection, privacy, and accountability are additional priorities. In simulated teaching and clinical environments, students have identified risks related to security, confidentiality, and the absence of updating or navigation. These risks necessitate clear rules regarding use, consent, and technical limits [35]. At the institutional level, data minimization policies, AI usage logs, and citation and contribution declaration guidelines are recommended, in addition to ethical oversight [19,30]. Students and faculty have expressed a need for clear rules, transparency regarding AI use, and authorship criteria, along with explicit policies and procedures at the course and institutional levels [19,30].

It is also advisable to establish a cycle of implementation and continuous evaluation. Trials indicate 'acceptable-marginal' usability and perceived benefits but underscore the need for frameworks of good practice, monitoring, and periodic review [30]. Institutions must have ethical frameworks, AI detection systems, and policies that regulate its responsible use [19-21,25,29-31]. They must also have inclusive and transparent strategies that address student concerns about possible sanctions when clear guidelines do not exist [32].

Discussion

ChatGPT is a large language model system by OpenAI that generates text via a conversational interface. Since its November 2022 launch, it reached approximately 100 million users within weeks, becoming the fastest-growing consumer app [27]. In undergraduate health sciences, adoption has been rapid: surveys report that ChatGPT is the most commonly used chat-AI among pharmacy students (82.8%) for assignments and studying [29]. Given this widespread uptake, universities are pivoting from technical capability to educational integration to ensure sound, ethical use in curricula.

The impact of ChatGPT depends on the academic field. In highly structured disciplines such as translation, academic writing, closed-ended problem solving, and programming, the tool has been seamlessly integrated and is valued for its immediate accuracy and time savings. This phenomenon has been observed in studies related to languages, computer science, and engineering [11]. In degrees with highly structured tasks, ensuring simplicity, visibility, and reliability is usually sufficient to encourage adoption [36]. The applications of generative AI are often concentrated in environments where tasks are repetitive and the added value lies in accelerating routine processes [37]. Deng et al. highlighted that the area with the highest usage was languages, due to their ability to explain, translate, and correct. In contrast, mathematics and law appeared less frequently and showed more irregular results, influenced by hallucinations or a reliance on memorized solutions rather than genuine understanding [38].

According to multiple studies, ChatGPT has been shown to produce information that is vague, outdated, or even fabricated. This information is often accepted uncritically by students, particularly in technical areas with high terminological demands and significant reliance on guidelines and protocols [19,20,25-27,34,35]. At the undergraduate level, the primary risks are hallucinations, dependency, and plagiarism (affecting the development of clinical foundations). At the graduate level and in healthcare settings, the critical issues are ethical and legal (privacy, bias, authorship, and responsibility). At both levels, the recommendation converges: human-in-the-loop, AI literacy, and transparent evaluation of the model and prompts [36].

In contrast, in health sciences, learning is structured around authentic tasks with potential consequences for patient safety. These tasks include building clinical judgment, prioritizing diagnoses, writing clinical notes, and communicating with people in sensitive situations. Therefore, the use of ChatGPT cannot be limited to making studying easier or faster. To ensure the responsible adoption of AI, it is essential to implement systematic teacher supervision, ensure the traceability of each interaction, and enhance the protection of sensitive data. Evaluation criteria should be in place to weigh the quality of reasoning and safety over mere efficiency [36,39,40]. In the field of undergraduate health sciences, trust and governance are paramount due to the sensitivity of the data and its impact on clinical reasoning and patient safety [18,19,22,35]. Therefore, before discussing ease or usefulness, it is advisable to ensure closed environments, anonymization of cases, and systematic human review [19,30].

Furthermore, the integration of the tool varies across healthcare settings. In areas such as obstetrics and gynecology, there is a research gap and a lack of empirical validation on the performance and detection of AI-generated content. This suggests the need to develop specific operational guidelines and quality controls [10]. Student confidence is more strongly influenced by the ability to verify the practical application of the tool in clinical decision-making than by generic demonstrations. This verification process includes clear limits, explicit justifications, and human review before the utilization of the tool in simulated practices or evaluations [39].

In 2025, Deng et al. conducted a review assessing whether ChatGPT enhances the academic performance, motivation, and attitudes of students and stimulates higher-order thinking and reduces mental effort, with no consistent changes in self-efficacy [38]. However, other studies have identified potential concerns regarding the overreliance on ChatGPT, including the threat to critical thinking, independent research, and analytical reasoning skills. Some students have expressed concerns that the use of ChatGPT may encourage laziness, reduce academic effort, and stifle creativity [18,35].

In the context of undergraduate health studies, student perceptions are favorable, though concerns regarding verification and reliability have emerged, underscoring the necessity to enhance the accuracy, traceability, and alignment with specific educational requirements [32]. In graduate and continuing health education, the focus shifts from ease of use to governance (explainability, privacy, and ethical-legal framework), with improvements in literacy after specific clinical courses [39]. Moreover, research on higher education positions generative AI as a tool for personalization and automation in contexts involving repetitive tasks, enabling rapid progress in areas outside of health [8,11]. This pattern is particularly pronounced in technical areas that require precise language, have many variants, and must adhere to specific guidelines and protocols for each discipline [21,26,27].

Personalization of learning emerges as one of the most consistent benefits in higher education. Adapting content, pace, and support improves autonomy and allows for early detection of disengagement to activate tutoring, alternative routes, and micro-feedback. This approach involves shifting the focus from grading at the end of the process to intervening during it. This strategy has the potential to reduce gaps and strengthen self-regulation [12]. In health sciences, personalization is only defensible if it can be audited through a log of prompts, mandatory anonymization of cases, and a minimum technical report supervised by individuals who document design and evaluation. This enhanced traceability transforms personalization into a verifiable clinical framework, thereby facilitating continuous enhancement, consistent with the guidelines for transparency, faculty oversight, and governance outlined for health education [36,39].

Faculty and students have repeatedly emphasized that AI cannot replace the value of human interaction in clinical teaching or professional identity development [20,34]. These concerns align with broader pedagogical critiques that AI tools can promote superficial learning if not integrated responsibly. Concerns also included ethical issues, lack of academic honesty, and fears that faculty would oppose or penalize the use of AI. These factors contribute to a complex and sometimes conflicted adoption landscape [32]. Many proposals for AI in higher education presuppose stable infrastructures, high digital literacy, and mature regulatory and data frameworks. However, these assumptions do not reflect the reality of institutions with technological or linguistic gaps. The risk is associated with two main factors: overestimating benefits and underestimating costs and damage in environments characterized by low resource availability and high cultural diversity [11].

Faculty have reported a lack of technical expertise and a fear of replacement, which has led to a reluctance to embrace change. To correct this, specific faculty training would be necessary, ensuring that AI does not replace academic and clinical work. In health sciences, this involves co-designing with clinical teachers and healthcare services, incorporating ethical and patient safety assessments, and reinforcing continuous professional development in AI [8,12].

Another aspect detected is the presence of cultural biases, which are not accidental. These biases derive from an unbalanced training corpus, predominantly in English, with an overrepresentation of Western sources. These aspects are internalized and reproduced by the model [18]. In the fields of education and health, this can perpetuate centralized viewpoints, render local practices and frameworks invisible, and generate decontextualized recommendations [21]. The literature indicates that if training data contains stereotypes or is biased, the system will likely replicate biases against marginalized communities [19]. Language models such as ChatGPT demonstrate inconsistent performance depending on language, country, and local norms. Sallam et al. documented that the tool performed better on a pharmaceutical exam in English than in its Chinese version. This finding indicates that multilingual validation and culturally sensitive studies are necessary before generalizing [40]. In clinical-educational practice, this bias manifests in responses that are not adapted to the legal or health frameworks of a specific country, creating an operational bias [36]. The literature also cautions that training sets can perpetuate inequalities, necessitating that students learn to identify and mitigate biases as part of their AI literacy training [39].

That is why efforts must be made to ensure equity and inclusion, both in terms of access and cultural biases. Infrastructure and training gaps hinder the equitable implementation of chatbots. Additionally, there are regional variations in perceptions of the potential of chatbots and concerns about reduced human interaction, which necessitate pedagogical support and equitable resources [23,31]. Governance frameworks should consider funding for access, support for underserved groups, and audits of biases in content and performance [19], as well as clear policies on mandatory disclosure of AI use and authorship criteria, along with guidelines on equity and accessibility [19,30].

The risks associated with cultural bias vary depending on the level of education. At the undergraduate level, the regular use of ChatGPT for summaries or conceptual questions is more affected by technical jargon or language than by local norms. In contrast, at the graduate and residency levels, where national clinical guidelines, protocols, and specific cultural contexts are in use, the risk of a non-contextualized response affecting patient safety is higher. A recent study in neuroscience revealed significant errors in advanced content, emphasizing the importance of human supervision, particularly at higher levels [41].

The digital divide continues to be a significant challenge. Crompton and Burke emphasize that the equity of educational AI is contingent on devices, connectivity, and digital literacy [11]. Similarly, Cortés et al. warn that without investment in infrastructure, inclusion policies, and international consortia, there is a risk of overestimating benefits in contexts of high cultural diversity and low resource availability [8]. Proposals such as regional quotas, North-South co-design, and the publication of open data and prompts seek to shift the agenda from mere technology transfer to the co-production of contextualized evidence [8,11].

The integration of ChatGPT in clinical education brings to light significant concerns regarding confidentiality, algorithmic transparency, and the potential for misusing patient data [20,21,31]. As the model is not able to explain its reasoning or verify sources, relying on its results could potentially compromise evidence-based decision-making [21].

The most frequently cited problem in the literature is the lack of ethical considerations. 40.9% of reviews emphasize the need for explicit attention to two key areas: ethics as a subject of study (biases, social impact) and research conduct (consent, data storage, transparency in evidence synthesis). Bond et al. identify specific gaps, including the insufficient treatment of privacy in primary studies, the need for ethical frameworks, and the need to integrate AI ethics into university curricula. Even poor practices are identified in reviews, such as incorrect citations of PRISMA and plagiarism, highlighting the necessity for more methodological approaches. In the field of health, it has been noted that a lack of AI literacy has the potential to transfer decision-making authority from professionals to systems, provided it is not managed effectively. Therefore, the focus should be on investments, training, and policies that position AI as a complement, not a substitute [12].

Cortés et al. propose the establishment of committees, ethical guidelines, and institutional protocols for the responsible use of AI. These guidelines would align personalization not only with efficiency or motivation but also with clinical reasoning and patient safety [8]. In practice, this means that the scope of ChatGPT should be limited to low-risk educational tasks, with comprehensive supervision by the teacher before it is used in evaluative or simulated contexts. This principle ensures that AI functions as scaffolding, but that responsibility for clinical judgment, evaluation, and safety remains in human hands [36,39].

In line with early systematic reviews, integration should be cautious and guided by policy. There should be explicit guidance on authorship, verification, and data protection to mitigate misuse and overdependence [36]. In terms of privacy and security, it is essential to implement clear policies, ensure transparency and explainability criteria, and establish a well-defined legal basis for the processing of personal data [8].

It is advisable for academic institutions to adopt policies that clearly define the circumstances under which ChatGPT can be utilized, differentiate between technical support and co-authorship, and prohibit any uses that contravene academic integrity [9]. Ray emphasizes that even in specialties with limited exploration, such as obstetrics and gynecology, the integration of ChatGPT necessitates addressing gaps in information validation and establishing evaluation and utilization protocols to ensure reliable outcomes in educational settings [10].

Limitations

This narrative review has several limitations that should be acknowledged. First, the review included only studies published between January 2023 and March 27, 2025. While this ensures the studies are relevant and current, it may have excluded earlier foundational work on generative AI in education. Second, the review focused exclusively on undergraduate health sciences education, omitting potentially valuable insights from postgraduate training, interprofessional education, and continuing professional development. This narrowed scope may have limited the generalizability of findings to other levels of healthcare education.

Despite the rigorous selection process that was used, which included keyword filtering and AI-assisted tools (Rayyan and ASReview), the final analysis was limited to 18 documents. This relatively small sample size may not fully capture the diversity of perspectives and implementations across regions and educational settings.

The narrative design of this review adheres to the criteria established in the scale for the quality assessment of narrative review articles (SANRA) [42]. Consequently, the interpretation of the data was thematic and descriptive, rather than statistical, which may introduce subjectivity. To mitigate potential bias, a structured quality assessment was applied to all included primary studies using the validated Mixed Methods Appraisal Tool (MMAT). This approach enabled a consistent evaluation of the diverse study designs and helped to ensure methodological rigor.

Furthermore, the initial search strategy in several databases retrieved a high volume of documents across a wide range of disciplines. Consequently, the selection had to be limited to subareas specifically related to health sciences, potentially excluding interdisciplinary perspectives relevant to the topic.

Moreover, the majority of included studies rely on cross-sectional designs and self-reported perceptions, which are inherently vulnerable to recall bias, social desirability bias, and lack of longitudinal insight. As a result, the findings may reflect short-term attitudes rather than sustained behavioral change. While the review incorporates data from diverse countries and educational systems, this international variability, though valuable in capturing a broad range of perspectives, also introduces heterogeneity in study design, cultural context, and technological infrastructure. This limits the comparability of findings and may affect the consistency of conclusions across settings.

Although this study is a narrative review, its analysis of the results is not quantitative. The presence of duplicate references among the reviewed documents could influence the perception of the breadth of the available literature. This consideration was taken into account during the interpretation of the evidence, ensuring that independent weight was not assigned to these repeated references.

Finally, the rapid evolution of generative AI tools like ChatGPT means that the findings may quickly become outdated. Technologies, educational policies, and ethical frameworks surrounding AI use in classrooms are constantly evolving, which limits the long-term generalizability of this review's conclusions.

Future research

As generative AI, particularly ChatGPT, becomes more integrated into our daily lives, especially in higher education, there is an urgent need for clear regulations backed by institutions. Universities must develop formal policies that promote the responsible use of AI in all academic disciplines, ensuring alignment with pedagogical objectives and academic integrity standards. In addition to regulation, there is a high demand for training programs tailored to students and faculty. These programs should provide users with the technical skills necessary to effectively use AI tools while fostering awareness of their limitations, ethical implications, and responsible use and data protection.

It is equally important to incorporate ethical principles into all aspects of AI use in education. Issues such as data privacy, security, and plagiarism prevention must be systematically addressed through institutional protocols and academic codes of conduct. ChatGPT and similar tools should be viewed as complementary resources that require responsible guidance and informed supervision, not as substitutes for human reasoning. Adopting these measures will ensure that AI enhances rather than undermines the integrity, inclusivity, and quality of higher education in the health sciences. Learning to apply generative AI in university training programs will enable future health professionals to develop useful tools for serving society.

## Conclusions

ChatGPT offers transformative potential as a support tool in university-level health sciences education. It is already widely used for academic writing, clinical simulations, and personalized learning across multiple disciplines. However, its integration also presents significant challenges, including risks to academic integrity, a lack of emotional intelligence, and gaps in AI literacy. Despite these concerns, ChatGPT is especially promising in areas such as curriculum design, diagnostic training, and inclusive education. To maximize its benefits, improvements are needed in accuracy, ethics, and pedagogical alignment. A structured, ethically guided, and human-centered approach is essential to ensure ChatGPT complements, rather than compromises, educational quality and professional development in the health sciences.

## Appendices

Appendix A

Tables 5-8 feature item-level judgments that are collated by design for the primary studies included in this review.

**Table 5:** Risk of bias assessment for qualitative studies

First Author and year	Are there clear research questions?	Do the collected data help address the research questions?	Is the qualitative approach appropriate to answer the research question?	Are the qualitative data collection methods adequate to address the research question?	Are the findings adequately derived from the data?	Is the interpretation of results sufficiently substantiated by data?	Is there coherence between qualitative data sources, collection, analysis, and interpretation?	Bias summary
Mohammed et al. (2024) [25]	Yes	Yes	Yes	Yes	Yes	Yes	Yes	7/7
Sarıkahya et al. (2025) [33]	Yes	Yes	Yes	Yes	Yes	Yes	Yes	7/7
Summers et al. (2024) [34]	Yes	Yes	Yes	Yes	Yes	Yes	Yes	7/7
Ahmed et al. (2024) [35]	Yes	Yes	Yes	Yes	Yes	Yes	Yes	7/7

**Table 6:** Risk of bias assessment for RCT RCT: Randomized controlled trial

First Author and year	Are there clear research questions?	Do the collected data help address the research questions?	Is randomization appropriately performed?	Are the groups comparable at baseline?	Are there complete outcome data?	Are outcome assessors blinded to the intervention provided?	Did the participants adhere to the assigned intervention?	Bias summary
Veras et al. (2024) [30]	Yes	Yes	Yes	Yes	Yes	No	No	5/7

**Table 7:** Risk of bias assessment for quantitative descriptive studies

First Author and year	Are there clear research questions?	Do the collected data help address the research questions?	Is the sampling strategy relevant to address the research question?	Is the sample representative of the target population?	Are the measurements appropriate?	Is the risk of nonresponse bias low?	Is the statistical analysis appropriate to answer the research question?	Bias summary
Salama et al. (2025) [23]	Yes	Yes	No	No	Yes	No	Yes	4/7
Oluwadiya et al. (2023) [24]	Yes	Yes	No	No	Yes	No	Yes	4/7
Song et al. (2024) [26]	Yes	Yes	No	No	Yes	No	Yes	4/7
Totlis et al. (2023) [27]	Yes	Yes	N/A	N/A	Yes	Yes	Yes	5/7
Cherrez-Ojeda et al. (2024) [28]	Yes	Yes	Yes	Partial	Yes	Yes	Yes	6.5/7
Orok et al. (2024) [29]	Yes	Yes	Yes	Yes	Yes	Yes	Yes	7/7
Uribe et al. (2024) [31]	Yes	Yes	Yes	Yes	Yes	Yes	Yes	7/7

**Table 8:** Risk of bias assessment for mixed-methods studies

First author and year	Are there clear research questions?	Do the collected data help address the research questions?	Is there an adequate rationale for using a mixed-methods design to address the research question?	Are the different components of the study effectively integrated to answer the research question?	Are the outputs of the integration of qualitative and quantitative components adequately interpreted?	Are divergences and inconsistencies between quantitative and qualitative results adequately addressed?	Do the different components of the study adhere to the quality criteria of each tradition of the methods involved?	Bias summary
Moskovich & Rozani (2025) [32]	Yes	Yes	Yes	Yes	Yes	Yes	Yes	7/7

Appendix B

Table 9 features the ratings for the reviewed articles.

**Table 9:** Risk of bias assessment for the reviewed articles

First author and year	Is the objective or research question clearly stated and justified?	Is the literature search strategy described in sufficient detail?	Are the inclusion and exclusion criteria clearly defined and appropriate for the objective?	Is the selection process of sources transparent?	Are the data extraction and synthesis methods described and appropriate for the type of review?	Are the findings organized and presented clearly, with consistent structure?	Does the review acknowledge its limitations?	Bias summary
Sahu et al. (2023) [18]	Yes	Partial	No	No	Partial	Yes	Yes	4/7
Hamad et al. (2024) [19]	Yes	Yes	Yes	Yes	Yes	Yes	Yes	7/7
Xu et al. (2024) [20]	Yes	Partial	No	No	Yes	Yes	Yes	4.5/7
Shorey et al. (2024) [21]	Yes	Yes	Yes	Yes	Yes	Yes	Partial	6.5/7
Gunawan et al. (2024) [22]	Yes	Yes	Yes	Yes	Yes	Yes	Yes	7/7
